# The value of breast MRI in high‐risk patients with newly diagnosed breast cancer to exclude invasive disease in the contralateral prophylactic mastectomy: Is there a role to choose wisely patients for sentinel node biopsy?

**DOI:** 10.1002/cam4.663

**Published:** 2016-03-18

**Authors:** Vivianne Freitas, Pavel Crystal, Supriya R. Kulkarni, Sandeep Ghai, Karina Bukhanov, Jaime Escallon, Anabel M. Scaranelo

**Affiliations:** ^1^ Department of Medical ImagingMount Sinai Hospital and University Health NetworkUniversity of TorontoTorontoOntarioCanada; ^2^ Department of SurgeryUniversity of TorontoTorontoOntarioCanada

**Keywords:** Breast cancer, breast MRI, contralateral prophylactic mastectomy

## Abstract

The aim of this study was to evaluate the presence of clinically and mammographically occult disease using breast MRI in a cohort of cancer patients undergoing contralateral prophylactic mastectomy (CPM) and the utmost indication of axillary assessment (sentinel node biopsy (SLNB)) for this side. A retrospective review of patients with unilateral invasive breast cancer or ductal carcinoma in situ (DCIS) from institutional MRI registry data (2004–2010) was conducted. Characteristics of patients undergoing CPM with breast MRI obtained less than 6 month before surgery were evaluated. A total of 2322 consecutive patients diagnosed with DCIS or stage I to III infiltrating breast cancer underwent preoperative breast MRI. Of these, 1376 patients (59.2%) had contralateral clinical breast exam and mammography without abnormalities; and 116 patients (4.9%) underwent CPM (28 excluded patients had breast MRI more than 6 months before CPM). The mean age of the 88 patients was 49 years (range 28–76 years). Two (2.3%) DCIS identified on surgical pathology specimen were not depicted by MRI and the 5 mm T1N0 invasive cancer (1.1%) was identified on MRI. Preoperative MRI showed 95% accuracy to demonstrate absence of occult disease with negative predicted value (NPV) of 98% (95% CI: 91.64–99.64%). Occult disease was present in 3.4% of CPM. MRI accurately identified the case of invasive cancer in this cohort. The high negative predictive value suggests that MRI can be used to select patients without consideration of SLNB for the contralateral side.

## Introduction

Prophylactic mastectomy (PM) reduces the risk of breast cancer in 90–95% if performed as a surgical preventive strategy in women at higher risk of developing breast cancer 1, 2, 3. Women with a personal history of breast cancer are considered to be at higher risk for developing contralateral breast cancer with the prevalence of synchronous cancer ranging from approximately 1–3% and up to 10% for the contralateral cancer detected in the follow‐up 4. Possibly for this reason some women opt for surgical removal of their breasts and data indicate that the use of contralateral PM (CPM) in women diagnosed with unilateral breast cancer more than doubled within the last 10 years 5.

Occult carcinoma has been reported in 3–9% of CPM specimens 6, 7, 8. In those cases the assessment of the lymph nodes becomes necessary and this is the reason why some surgeons recommend considering sentinel node biopsy (SLNB) as a routine in CPM because the only alternative if an invasive cancer is found is to do axillary lymph node dissection (ALND). Currently SLNB has been an accepted method for identifying metastasis in patients with early stage of breast carcinoma with significantly lower morbidity than ALND 9, 10, 11. However, the role of the utility of SLNB in patients undergoing PM is still controversial 6, 7, 12, 13, 14, 15, 16, 17.

In addition, it is well known that MRI is capable to detect small cancers in high‐risk population 18, 19, 20, 21, 22. Furthermore, it has been shown that MRI may detect contralateral mammographically and clinically occult breast cancer in 3–18% of women with newly diagnosed breast cancer 23, 24. The sensitivity of MRI in detecting invasive cancers (IC) ranges from 80% to 100% 20 and with ongoing data demonstrating that MRI is an important screening tool it is expected that preoperative MRI could potentially reduce the number of unsuspected malignancies found at CPM specimens. However, there are sparse data to assess the need of axillary staging (most of the times using SLNB) in women undergoing CPM 13, 15. For this reason, we aimed to evaluate the presence of clinically and mammographically occult disease using breast MRI in a cohort of cancer patients undergoing CPM and the utmost indication of axillary assessment SLNB for this side.

## Material and Methods

### Population

The Institutional Research Ethics Board approved this retrospective population‐based cohort study linked data from imaging‐pathology database available through a Canadian tertiary hospital (Princess Margaret Hospital from University Health Network) that is affiliated to the University of Toronto to capture patients diagnosed with ductal carcinoma in situ (DCIS) or stage I to III infiltrating breast cancer that underwent preoperative breast MRI from January 2004 to December 2010.

The inclusion criteria were patients with recently diagnosed of breast cancer that underwent CPM with normal contralateral screening tests including clinical breast examination, and mammography. To identify factors associated to imaging findings, patient‐level data were collected for breast MRI tests that do not exceeded more than 3 months after mammography and no more than 6 months before the CPM.

### Imaging analysis

MRI examinations were performed on 1.5T systems (Signa Excite, General Electric Medical Systems, Milwaukee, WI or Espree or Avanto; Siemens Healthcare, Erlangen, Germany) and a 3.0T system (Verio, Siemens Healthcare, Erlangen, Germany) with a standard bilateral dedicated breast coil. Breast MRI protocols were complying with quality standards of American College of Radiology on dates the exams were performed. Preoperative exams prospectively reported were reviewed and classified according to the Breast Imaging Reporting and Data System (BI‐RADS) lexicon 25: categories BI‐RADS 1, 2 and 3 were considered negative and the categories 4 and 5 were considered as positive tests. In the setting of CPM short‐term follow‐up is not feasible 26, therefore after the MRI‐induced work‐up performed (SLU + USCNB or MRI‐guided biopsy) all exams classified as BI‐RADS 3 were considered as negative examinations for statistical analysis purposes and the category BI‐RADS 0 was not used for this study. Indeed, exams initially scored as BIRADS 0 were reviewed in consensus by 2 board certified and fellowship trained breast radiologists (XX, 18 years of experience; and XXX, 11 years of experience) blinded to pathology results and a final category from 1 to 5 was assigned based on the results of MRI‐induced work‐up.

### Surgery and pathology analysis

All patients underwent CPM at the same time of the index breast surgery or after the curative surgery based on patient and surgeon preferences. All mastectomy specimens including the ones of the CPM were prospectively assessed in a standardized manner by one of the three subspecialty trained and CAP (College of American Pathologists) certified breast pathologists that have a range of 15–30 years of expertise in breast pathology. The histological findings in the CPM specimen were reviewed and classified for this study as: (1) benign (e.g., fibroadenoma; fibrocystic disease, sclerosing adenosis, and benign breast tissue without atypia); (2) high‐risk (e.g., atypical lobular hyperplasia – ALH; atypical ductal hyperplasia – ADH; lobular carcinoma in situ – LCIS; flat epithelial atypia – FEA); and (3) malignant (e.g., DCIS and invasive carcinoma).

### Statistical analysis

Descriptive statistics for the variables of interest (mean age, BI‐RADS lexicon descriptors, and lesion size) were calculated. Continuous variables were described using mean ± SD and categorical variables using frequency and percentage. MRI accuracies measures including sensitivity, specificity, positive and negative predictive values were calculated on a per‐patient basis. A *P*‐value < 0.05 was considered statistically significant.

## Results

A total of 2322 consecutive patients diagnosed with DCIS or stage I to III infiltrating breast cancer underwent preoperative breast MRI. Of these, 1376 patients (59.2%) had normal contralateral clinical breast exam and mammography; and 116 patients (4.9%) underwent CPM (28 patients had breast MRI more than 6 months before CPM). Of 1376 patients with normal contralateral breast, 88 patients (6.4%) had at least one breast MRI exam not exceeding more than 6 months before the date of the CPM. Of those 88 patients (age ranged 28–76 years; mean 50 years), 64% of women with CPM had some degree of increased risk for developing breast cancer: 19 (34%) patients were previously tested positive for BRCA mutation; seven (12%) patients had prior history chest radiation; and 30 (54%) patients had family history of breast cancer in 1st degree relatives. Patient cohort clinical data are summarized on Table 1.

**Table 1:** Distribution by age of 88 patients that underwent contralateral prophylactic mastectomy (CPM) based on risk factors

CPM cohort's age	Total number(*n *=* *88, 100%)	BRCA mutation carriers(*n *=* *19, 34%)	Mantle radiation(*n *=* *7, 12%)	Family history of breast cancer(*n *=* *30, 54%)
Age < 40	16 (18.2)	4 (21)	4 (57.2)	3 (10)
Age 40–49	33 (37.5)	5 (27)	2 (28.6)	15 (50)
Age 50–59	22 (25)	6 (31)	–	5 (16.7)
Age 60–69	15 (17)	3 (16)	1 (14.2)	7 (23.3)
Age > 70	2 (2.3)	1 (5)	–	–

**Figure 1:**
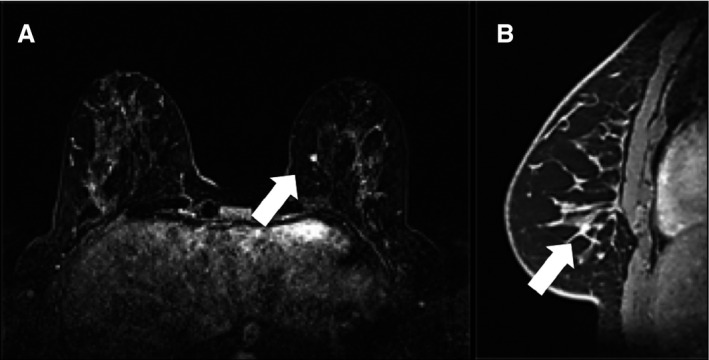
Preoperative breast MRI of a 52 years‐old woman, without family history of breast cancer with a newly diagnosed right breast cancer (not seen on the images). (A) Dynamic contrast‐enhanced breast MRI on the axial plane with postprocessed subtracted T1‐weighted images with fat saturation obtained 2 min after contrast injection and (B) Dynamic contrast‐enhanced breast MRI with postprocessed sagittal plane of subtracted T1‐weighted images with fat saturation obtained 2 min after contrast injection showing the focus of enhancement in the inner part of the left breast.

The surgical pathology results of the CPM specimen for all patients are shown in Table 2. Breast malignancy was diagnosed in three mastectomy specimens of the 88 patients (disease prevalence of 3.4%; 95% CI: 0.75–9.65%), where one specimen showed invasive cancer measuring 5 mm stage 1 (pT1a) tumor that was identified as a 4.8 mm focus of wash‐out enhancement in the BIRADS 4 category preoperative MRI (Fig. 1) from 2009; and the other two specimens demonstrated each one an intermediate grade in situ carcinoma, measuring 2.6 cm and 0.3 cm, respectively, that none were depicted by MRIs performed, respectively, August 2007 and November 2005.

**Table 2:** Histopathology findings in the 88 mastectomy specimens of the clinically negative contralateral breast

Histopathology	Number (*N *=* *88)
Benign^a^	53 (60.2%)
Atypical ductal hyperplasia (ADH)	6 (6.8%)
Atypical lobular hyperplasia (ALH)	15 (17%)
Lobular carcinoma in situ (LCIS)	4 (4.6%)
Flat epithelial atypia (FEA)	7 (8.0%)
Ductal carcinoma in situ (DCIS) Size 2.6 cm & 0.3 cm	2 (2.3%)
Invasive ductal carcinoma Size 0.5 cm	1 (1.1%)

a Benign lesions were described as fibroadenoma; fibrocystic disease, sclerosing adenosis and benign breast tissue without atypia.

Breast MRI results of the contralateral breast classified according to the BI‐RADS system are demonstrated in Table 3. The preoperative breast MRI with the MRI‐induced work‐up demonstrated 95% accuracy to demonstrate that the contralateral breast does not have occult disease with a high negative predicted value (NPV) of 98% (95% CI: 91.64–99.64%), 33% sensitivity, 97% specificity, 25% positive predictive value (PPV) (Table 4).

**Table 3:** Distribution of breast MRI classification of the contralateral breast according to breast imaging reporting and data system (BI‐RADS) lexicon and surgical pathology

MRI classification	Number (*N *=* *88)	Prophylactic mastectomy outcomes
BI‐RADS 1	30 (34%)	30 Benign/high‐risk results
BI‐RADS 2	43 (49%)	41 Benign/high‐risk results2 Malignant^a^ results ductal carcinoma in situ (DCIS)
BI‐RADS 3	11 (12.5%)	11 Benign/high‐risk results
BI‐RADS 4	4 (4.5%)	3 Benign/high‐risk results1 Malignant^a^ invasive cancers (IC)
BI‐RADS 5	0	__________

a Malignant results were one case of invasive ductal carcinoma measuring 5 mm and two cases of ductal carcinoma in situ.

**Table 4:** Distribution of the breast imaging reporting and data system (BI‐RADS) 4 Category of MRI results based on pathology outcomes

ACR BIRADS 4 category cases	Patient age (years)	MRI lesion/size (mm)	Interventional procedure	Core biopsy histopathology results	Pathology size (mm)	Surgical pathology results
Case 1	41	Focus/5	MRI‐guided vacuum‐ assisted biopsy	Benign	Benign (not reported)	Flat epithelialatypia
Case 2	36	Focal area of nonmass enhancement/6.5	US‐guided FNA	Papillary neoplasm	Benign (not reported)	Papilloma
Case 3	52	Focus/5	MRI‐guided needle localization	–	5	Invasive ductal carcinoma
Case 4	52	Mass/15	US‐guided core biopsy	Benign	7	Radial scar

## Discussion

Our results demonstrates 3.4% prevalence of occult malignancy in a consecutive cohort of high‐risk patients receiving breast MRI before undergoing CPM that was not different from other cohorts of women that underwent to a PM 6, 8. Indeed, MRI correctly identified the subcentimeter invasive cancer but did not have resolution to identified the two cases of intermediate grade DCIS that were done before 2008. Our findings are supported by several studies 18, 19, 20, 21, 22 where MRI sensitivity for detecting high‐grade DCIS as well invasive disease is up to 100% but the sensitivity of this method alone for detecting low‐to‐intermediate grade of DCIS is not so high. One may conclude that studies 18, 19, 20, 21, 22 elsewhere and our results used MRI technology that was standard of art for the time the tests were done, but not represent the current status of the better resolution equipments and dedicated breast coils that are available at the present moment and certainly may impact the detection of intermediate and low‐grade DCIS. Moreover, the clinical significance of in situ cancers not identified on contrast‐enhanced MR images but detected by pathologists on clinically healthy breasts at CPM specimens remains unknown because nobody truly knows if the areas of in situ disease would progress to have clinical impact or they would have entered in remission 27. Indeed, Fancellu et al. demonstrated in a recent meta‐analysis that preoperative MRI in women with DCIS is not associated with improvement in surgical outcomes 28.

The greatest strength of our study certainly is to confirm the highest MRI‐negative predictive value for IC in this cohort that was of 100%. The clinical impact is relevant when obtained by a noninvasive preoperative method as the SLNB is the standard procedure in patient with invasive disease. However, it is well known that the risk of axillary node involvement with metastatic disease in women undergoing CPM is overall low reported less than 1% 16. Therefore, the utility of SLNB during CPM remains controversial 6, 8, 12, 13, 14, 15, 16, 17. The argument favoring routine SLNB in conjunction with CPM is centered on the inability to perform SLNB surgery once a mastectomy has been performed, committing patients whom IC is found to ALND for lymph node stating, which is associated which greater morbidity when compared with SLNB 9, 10, 11. The argument against the use of SLNB during CPM is the complications associated with SLNB surgery that have been reported from randomized trials 10, 11 and although lower when compared with ALND those potential adverse effects should not be negligible 9, 10, 11. In this controversial setting, to know exactly what is the subset of patients for whom SLNB is recommended is advisable. The detection rates of carcinoma in the contralateral breast range from 0.2 to 1.0% on clinical examination and from 1 to 3% using mammography 29, 30, 31 where the use of MRI in women with newly diagnosed breast cancer demonstrates a detection rate of clinically occult breast cancer ranging from 3% to 18% 23, 24. Nevertheless, the literature is scanty and controversial when evaluating the role of MRI in the surgeon's decision to use SLNB in women undergoing CPM. McLaughlin et al. 15 concluded that the high negative predictive value implies an important role of MRI to select patients that SLNB could be avoided, which is agreement with our results. Nevertheless, Black et al. 13 concluded that MRI is neither practical nor cost effective in the PM setting.

The IC detected in our study was an early stage breast cancer as showed in other studies included in a meta‐analysis 32 of sentinel lymph node biopsy at the time of PM. Considering the conclusions of the Z0011 trial 11 that in patients with early stage breast cancer we could only observe axilla even if SLNB is positive instead to proceed ALND, we could argue that SLNB in the prophylactic setting would be considered as an “overtreatment” 13. Nevertheless, and despite of the possibility of a not required and excessive invasive axillary procedure, SNLB in the CPM scenario has being performed by many surgeons around the world without established guidelines specifically to this particular setting and we speculate if a negative MRI result is available that gives a high level of confidence to the surgeon that invasive cancer is not present if this could be a valuable tool to impact in surgeon's decision to avoid SNLB for the sake of less aggressive management to the patient 33.

Our results shows that from 88 patients only three (3.4%) performed an imaging‐guided biopsy based on MRI recommendation. The remainder patients went straight to CPM and for all of these three patients, the MRI biopsy was benign and it did not change in the decision of CPM. Other authors also demonstrated that in the CPM setting the patient will undergo to mastectomy independently of the biopsy result that was performed based on MRI recommendations 26. It is out of scope of this study analyzing which factor affect decision of performing CPM in women with newly diagnosed breast cancer. However, accordantly to a prior study 26 young women perceived of greater risk of breast cancer as well as patients with high‐risk factors as mutation carries, prior history of chest radiation and family history of breast cancer which could influence the decision maker of CPM. Besides that, following previous study 34, 35 index breast cancer characteristics, such tumor size, histology type and nodal status were not predictors of finding occult malignancy in the contralateral breast, avoiding us to make any association of primary tumor and contralateral occult breast malignancy. Nevertheless, Laronga and colleagues 16 concluded that patient with locally advanced primary breast cancer seems to be at risk of positive SLNB in the contralateral breast even with occult contralateral breast malignancy as part of crossover disease. Therefore, in this group of patients if CPM is performed, the SLNB may be suitable independently of MRI results.

One of the major limitations of this study is to be a retrospective one and consequently there was a random time to perform the CPM, which means that in some cases the CPM was performed at the same time of index breast surgery and in other cases after systemic treatment, including neoadjuvant chemotherapy which could impact the frequency of IC detected in the CPM specimens. The other limitation was not considering the cost of adding MRI in the decision maker of CPM which could impact the consistent use of this high cost method in a daily routine.

Then, accepting such limitations we could infer MRI as a reliable modality to select patients for CPM without SLNB based on the highest NPV results (100%) for invasive cancer detection. In conclusion a negative preoperative MRI may preclude SLNB in women undergoing PM limiting the potential morbidity of this procedure in those patients.

## Conflict of Interest

Supported by JDMI, University of Toronto, Toronto, ON, Canada. The work had no specific funding as well there are no financial disclosures from any author.
